# DGKZ Acts as a Potential Oncogene in Osteosarcoma Proliferation Through Its Possible Interaction With ERK1/2 and MYC Pathway

**DOI:** 10.3389/fonc.2018.00655

**Published:** 2019-01-04

**Authors:** Wenxi Yu, Lina Tang, Feng Lin, Yang Yao, Zan Shen

**Affiliations:** Department of Oncology, Affiliated Sixth People's Hospital, Shanghai Jiaotong University, Shanghai, China

**Keywords:** osteosarcoma, DGKZ, oncogene, ERK1/2, MYC

## Abstract

Osteosarcoma (OS) is one of the most common primary bone tumors in children and young adults. The majority of osteosarcoma patients have limited alternative therapeutic options and metastatic patients generally have a poor prognosis. Thus, it is important to explore novel effective therapeutic targets in the treatment of osteosarcoma. Diacylglycerol kinase zeta (DGKZ) is a recently identified gene potentially associated with certain human carcinogenesis. However, the role of DGKZ in proliferation of osteosarcoma is still unclear. In this study, DGKZ's expression was firstly investigated in OS tumor samples and correlated with poor outcome in OS patients. Silence of DGKZ by shRNA hampered osteosarcoma cell growth and promoted cell apoptosis *in vitro*. *In vivo*, DGKZ's knockout also suppressed xenograft tumor proliferation as determined by bioluminescence imaging and weight/volume measurements. Meanwhile, Affymetrix GeneChip and Ingenuity Pathway Analysis (IPA) revealed that DGKZ knockdown resulted in a decreased activity of MYC pathway, and several target genes expression in MYC pathway were altered, including CCND1, CDKN2B, CDK6, PCNA, and EGR1. Furthermore, immunoprecipitation coupled with mass spectrometry (IP-MS) analysis was used to identify proteins that interacted with DGKZ in OS cells and revealed ERK1/2, a key MYC-interactor, to associate with DGKZ. Together, our study demonstrated that DGKZ might act as an oncogene in osteosarcoma via its possible interaction with ERK1/2 and MYC pathway.

## Introduction

Osteosarcoma is the most common aggressive form of bone tumor occurs in children and young adults (1, 2). Traditional therapeutic approaches include local control of the primary lesion by surgery and/or chemotherapy, and treatment of disseminated disease with multiagent cytotoxic.

Chemotherapy (3), Somehow, patients those with metastatic or recurrent disease have extremely poor survival rates even after normative chemotherapy and curative resection of the primary lesion (4–6). Hence, there is urgent need for development of new therapeutic approaches and a further investigation in proliferation of osteosarcoma. However, progression of osteosarcoma is associated with numerous genes with diverse genomic alterations and the molecular carcinogenesis of osteosarcoma has not been fully elucidated (7).

Diacylglycerol (DG) is an important second messenger involved in a variety of cellular responses including proliferation, differentiation, motility, and secretion (8, 9) which could activate various effector proteins. Intracellular DAG levels have been shown to play a vital role in cellular growth responses (10). Thus, levels of DG should be maintained strictly to keep cellular environment within a physiological range. Diacylglycerol kinases (DGK) are a class of enzymes that convert DAG into PA and act as key modulators of DG levels (11). Recent studies have found 10 isozymes of DGK, each showing unique tissue expression and distinct subcellular localization (12–15). Among the 10 isoforms, diacylglycerol kinase zeta (DGKZ) is the only one that contains a nuclear localization signal (16, 17) and has proven to be associated with various physiological or pathophysiological signaling pathways (16, 18–25). Recent studies have proven that in colon cancer and gliomas, DGKZ plays an key role in oncogenesis. (26–28). Additionally, DGKZ also showed its function in regulating bone homeostasis through osteoclasts via modulation of c-Fos (20), which is a key regulator in proliferation of osteosarcoma (29–32). However, the potential role of DGKZ in oncogenesis of osteosarcoma is still unclear.

In our study, DGKZ's expression was upregulated in osteosarcoma tissues and correlated with patients' poor prognosis. *In vitro*, DGKZ knockdown impaired proliferation and promoted apoptosis in osteosarcoma cells. *In vivo*, DGKZ-silencing xenografts in nude mice formed smaller tumors while compared with control group. Additionally, microarray and IPA analyses demonstrated a alternation in expression of signaling-related genes in osteosarcoma cells and verified MYC pathway as a direct downstream target of DGKZ. In addition, association between DGKZ and ERK1/2, a key MYC-interactor, was verified through immunoprecipitation-mass spectrometry (IP-MS) approach. These results suggest a potential interaction between DGKZ and ERK1/2-MYC pathway might play an important role in promotion of osteosarcoma, indicating that interfering with function or expression of this interaction may be a potential route to block the invasiveness of osteosarcoma.

## Materials and Methods

### Ethics Statement

The Ethics Committees of Affiliated Sixth People's Hospital, Shanghai Jiaotong University has approved all studies about human participants. Informed and written consents were obtained from all patients or their advisers according to ethics committee guidelines.

### Patient Samples and Follow-Up

Tumor specimens from 80 patients suffered from IIB limb osteosarcoma between 2012 and 2015 and 24 cases of normal bone tissues as control group were collected from Affiliated Sixth People's Hospital, Shanghai Jiaotong University for this study. The normal bone tissues were resected within at least 5 cm to the margin of tumor while definitive surgery was administrated. After surgical resection, all the osteosarcoma and normal bone tissues were frozen within 30 min. All patients were treated with combination chemotherapy which consist high doses of methotrexate, cisplatin, ifosfamide, and doxorubicin, then underwent local resection of the primary tumor, followed by adequate cycles of adjuvant chemotherapy. After completion of chemotherapy,

patients were followed every 3 months for 4 years and then every 6 months for 10 years.

### Immunohistochemical Staining of DGKZ in OS Samples

Paraffin sections were treated with hydrogen peroxide to inactivate endogenous peroxidases. Antigen retrieval was performed in a microwave in 10 mmol/L citrate buffer at pH 6.0. Sections were then incubated in anti-DGKZ antibody overnight at 4°C, and a secondary antibody was used to detect protein expression (27). For each run of immunohistochemistry, appropriate positive and negative controls were performed. Immunohistochemistry staining was assessed by two blinded independent pathological observers who were blinded to patient identity. Percentage of osteosarcoma cells stained positive for DGKZ was recorded, with 0 denoting none of osteosarcoma cells stained, 1 denoting 1–25% of osteosarcoma cells stained, 2 denoting 26–50% of osteosarcoma cells stained, 3 denoting 51–75% of osteosarcoma cells stained, and 4 denoting 76–100% of osteosarcoma cells stained. Intensity of nuclear/cytoplasm/cytomembrane staining on a scale was also recorded, with 0 denoting no staining, 1 denoting slight/weak staining, 2 denoting strong staining; and 3 denoting intense staining. Score was calculated as percentage of cells stained positive multiplied by intensity of staining, and was defined as positive (score above 6) or negative(score ≤6).

### Cells Lines and Cell Culture Conditions

Osteosarcoma cell lines that included SaoS-2, U-2OS, HOS, MG-63, human normal osteoblasts hFOB1.19 cell line and human renal epithelial 293T cell line were obtained from China Center for Type Culture Collection (CCTCC, Shanghai, China). Cells were grown in Dulbecco's modified Eagle's medium, Eagle's minimal essential medium, DMEM-F12 growth medium, and McCoy's 5A medium mixed with 10.0% fetal bovine serum (FBS) that were purchased from Gibco (Gibco, USA). The cells were incubated overnight at 37°C in a 5% CO2 humidified environment. The cells were trypsinized when they reached 75% confluence and were then used for *in vitro* and *in vivo* studies.

### Quantitative Real-Time Reverse Transcriptase-Polymerase Chain Reaction

Quantitative real-time RT-PCR was performed in triplicate with an Applied Biosystems Prism 7,500 Fast Sequence Detection System using TaqMan universal PCR master mix according to the manufacture's protocol (Applied Biosystems Inc., Foster City, California, USA). TaqMan probes and primers were purchased from Applied Biosystems Inc. Levels of RNA expression were determined using the 7,500 Fast System SDS software package (version 1.3.1; Applied Biosystems Inc.) and GAPDH (glyceraldehyde 3-phosphate dehydrogenase) was used as a control for normalization. Primers used here were as follows: GAPDH for: 5′-TGACTTCAACAGCGACAC- -CCA-3′,GAPDH, reverse:5′-CACCCTGTTGCTGTAGCCAAA−3′, DGKZ for: 5′-AGCAAG–CAAGAAGAAGAAGAGG-3′, and DGKZ reverse:5′-GGATTGAGATACCAGAGGAAAGAC–3′. The relative DGKZ expression was normalized to GAPDH, and data analysis was conducted using the comparative CT method.

### DGKZ shRNA Design and Lentivirus Construction

Targeted shRNA was used to knockdown the expression of DGKZ in OS cells. Sets of lentiviral plasmids pGCSIL-GFP (GeneChem, Shanghai, China) with sequences targeting to DGKZ mRNA(sense, 5′-TCGCACAGGATGAGATTTATA-3′;antisense,5′-TATAAATCTCATC- CTGTGCGA-3′) and pGCSIL-GFP with non-targeting sequence as negative control were used for gene knockdown studies. Infectious shRNA-lentiviruses were packaged in 293T cells, purified and used to infect U2OS, Saos-2 and HOS cells for generation of stable transduced clones.

### Cell Proliferation Analysis

Cells in shCtrl and shDGKZ groups were plated in 96-well plates at a density of 2 × 10^3^ cells/well and incubated for 1–5 days. Each group contained three wells. At the end of incubation, 10 μl of 5 mg/mL MTT (Genview, USA) was added and incubated for 4 h at 37°C. The medium was removed and 150 μL DMSO was added. Absorbance was determined with an enzyme-linked immunosorbent assay reader at 595 nm. The cell proliferation curves were drawn according to the absorbance.

Cell proliferation was also recorded and accessed through counting viable cell number with Cellomics Array-ScanTM VTI HCS Reader (Thermo Scientific, Waltham, MA, USA)by manufacturer's instructions. Briefly speaking, cells were cultured at a density of 2^*^10^3^/well in 96-well plates under 37°C with 5% CO_2_. From the next day, cells with GFP were taken photos and counted each day. Cell proliferation was recorded consecutively for 5 days. Cell growth curves were drawn according to cell numbers.

### Cell Apoptosis Analysis

Cell apoptosis was assayed by staining with Annexin V-APC and detected by flowcytometry. Briefly, cells were washed twice with cold PBS and resuspended in 1 × binding buffer. Then 100 μl of solution (about 1 × 10^6^-1 × 10^7^ cells) was transferred to a 5 μl Annexin V-APC and 5 μl PI, and incubated for 15 min at room temperature in the dark. Cells were analyzed using flow cytometry. All experiments were performed in triplicate.

The activity of Caspases3/7 in OS cells was detected with Caspase-3/7 Assay Kit (Promega), following the manufacturer's instructions and cell fluorescence intensity at 499 nm was measured by ELISA Tablet counter for quantitative assessment.

### Assessment of Tumor Growth Inhibitory Effects of DGKZ -Silencing in a Xenograft Model

The mouse experiments and animal care procedures were approved by the Ethics Committee of Affiliated Sixth People's Hospital, Shanghai Jiaotong University. Four-week-old male BALB/c nude mice were obtained from Shanghai Laboratory Animal Center (Shanghai, China). HOS cells (4 × 10^6^) stably expressing DGKZ or shDGKZ were suspended in 150 μL of phosphate-buffered saline and inoculated subcutaneously in the right armpit region (*n* = 10 per group). After 4 weeks, the mice were sacrificed and the tumors were removed for analysis. Tumor volumes were calculated using the following formula six times during observation by Vernier calipers: 3.14/6 × y (length) × x^2^(width). After the mice were killed, the tumors were resected and weighted. In addition, before sacrificed, tumor growth in the living animals was monitored and quantified by luminescence levels. Bioluminescence was measured with the IVIS imaging system (Xenogen Corp., Alameda, CA, USA). All of the images were taken 10 min after intraperitoneal injection of luciferin (Sigma-Aldrich, St. Louis, MO, USA) of 150 mg/kg body weight, as a 60-s acquisition and 10 of binning. During image acquisition, mice were sedated continuously via inhalation of 3% isoflurane. Image analysis and bioluminescent quantification was performed using Living Image software (Xenogen Corp).

### Microarray Gene Expression Analysis

After infection with shDGKZ total RNA was extracted from HOS osteosarcoma cells, and 50–500 ng of RNA was used to generate biotin-modified amplified RNA (aRNA) using a GeneChip primeview human Kit (Affymetrix, USA). Data of gene expression was analyzed by GeneChip® PrimeView ™ Human Gene Expression Array (Affymetrix, USA; catalog # 901838) which contains probes for 36,000 genes and subsequent analysis was performed using the Affymetrix Expression Console (EC, version 1.1).For the microarray analysis, following filtering by flag signal, the raw data were processed and normalized. Differentially expressed genes were identified with *P* < 0.05 and fold changes >2. In addition, IPA (Ingenuity Pathway Analysis) was performed which provided more detailed information regarding the pathways in which the target genes were involved.

### Affinity Purification of DGKZ Interacting Complexes and LC-MS/MS Analysis

The DGKZ gene fused with a 3 × FLAG tag sequence (Sigma) at its 5′-end was amplified by PCR and inserted into GV367 lentiviral vector (Genechem, Shanghai, China), and the construct was named as p3 × FLAG-DGKZ. HOS cells were plated at the concentration of 4 × 10^5^ cells/well in 6-well plate, 2 ml of DMEM medium containing10 μl of 3 × FLAG-DGKZ or negative control lentivirus and 2 μl of polybrene(10 μg/μl) was added into the wells. The stable cells expressing 3 × FLAG-DGKZ was validated by immunoblotting using the specific antibodies. The 3 × FLAG-DGKZ interacting complex was purified using the anti-FLAG magnetic beads (Sigma) according to the manufacture's protocol. The procedure of in-gel trypsin digestion and peptide extraction was performed following the standard protocol. LC-MS analysis was performed on a Nano Acquity UPLC system (Waters Corporation, MA, U.S.) connected to a quadrupole-Orbitrap mass spectrometer (Q-Exactive) (Thermo Fisher Scientific, Bremen, Germany) equipped with an online nano-electrospray ion source. The Q-Exactive mass spectrometer was operated in the data-dependent mode to switch automatically between MS and MS/MS acquisition. Data from both scans were acquired in profile mode. The spectra were recorded with Xcalibursoftware.

The raw mass files generated by the Q-Exactive instrument were processed using Proteome Discoverer software (Thermo Scientific, version 1.4) integrated with the MASCOT (Matrix Science, London, UK; version 2.3.2) search engine for protein identification. Data were searched against the Human UniProtKB/Swiss-Prot database (Release 2017_02_19, 20185 entries).To reduce false positive identification results, a decoy database containing the reverse sequences was appended to the database.

### Co-immunoprecipitation and Immunoblotting

The procedure of protein isolation and immunoprecipitation was essentially the same as described in the purification of 3 × FLAG-DGKZ complexes for MS identification. The immunoprecipitated protein complexes were eluted by 3 × FLAG peptide. Samples were separated by SDS-PAGE and transferred on PVDF membrane (Millipore, 0.2 μm). The membranes were blocked with 5% non-fat milk and probed with specified primary antibody followed by incubation with the secondary antibody conjugated with horseradishperoxidase, and then the ECL substrate was added on membrane and exposed by ECL system.

### Statistical Analysis

Patient survival was analyzed by the log-rank test using SPSS 15.0 software. All experiments were performed in triplicates, and the data were expressed as the mean ± SD. Statistical analyses were conducted with Student's *t*-test. *P* < 0.05 was considered statistically significant.

## Results

### High Expression of DGKZ in Osteosarcoma Samples/Cells and Its Correlation With Poor Prognosis

**Figure 1:**
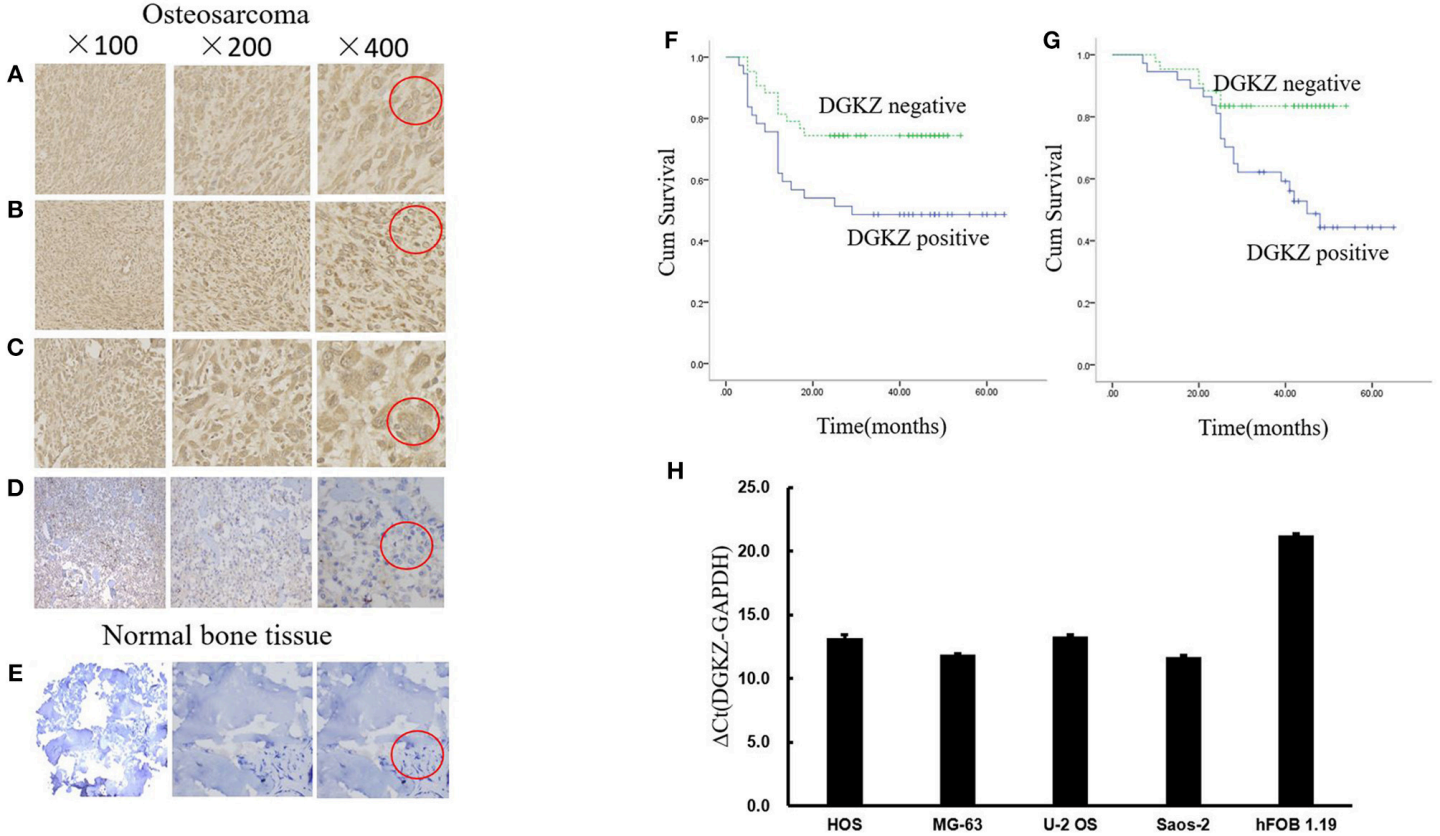
DGKZ expression in osteosarcoma samples and cells. **(A–C)** Representative images of osteosarcoma tissue with strong staining (brown) of DGKZ (Red circle represented characteristic positive position). **(D)** Osteosarcoma with negative DGKZ staining (Red circle represented characteristic negative position). **(E)** Normal bone tissue with negative DGKZ staining (Red circle represented characteristic negative position). **(F)** DGKZ expression is correlated with reduced progression-free survival in osteosarcoma patients. **(G)** DGKZ expression is correlated with reduced overall survival in osteosarcoma patients. **(H)** qPCR analysis of DGKZ expression in different osteosarcoma cell lines and human normal osteoblasts hFOB1.19.

To initially detect expression of DGKZ in osteosarcoma, we collected 80 osteosarcoma samples and 24 normal bone samples. Among 80 tumor samples, 37 osteosarcoma samples(46.2%) had high expression of DGKZ with a score defined as positive (above 6). Compared with osteosarcoma samples, DGKZ expression was absent in normal bone samples while all bone samples demonstrated a score defined as negative(≤6, Figures 1A–E). Additionally, DGKZ was detected to distribute in cytoplasm. Furthermore, The level of DGKZ expressed in four OS cell lines (SaoS-2, U-2OS, HOS, MG-63) was significantly higher than normal osteoblasts hFOB1.19 (Figure 1H).

No correlation was observed between age/gender/tumor location/pathological subtype and DGKZ expression (Table 1). DGKZ expression was associated with a poor prognosis in IIB limb osteosarcoma patients. According to data from follow-up, while evaluating progression-free survival, patients with DGKZ positive expression had a mean survival time of 36.7 months, whereas patients whose samples with DGKZ negative expression had a mean survival time of 42.9 months (*P* = 0.018, Figure 1F). Additionally, DGKZ expression was also associated with poor overall survival [mean, 44.7 months (positive) vs. 48.2 months (negative), *P* = 0.007, Figure 1G].

**Table 1:** Characteristics of 80 osteosarcoma patients.

	**DGKZ -positive**	**DGKZ -negative**
**GENDER**		
Male	26	29
Female	11	14
**AGE**		
≥18 years	22	25
<18 years	15	18
**HISTOTYPE OF TUMOR**		
Conventional	33	38
Other	4	5
**INITIAL TUMOR SITE**		
Femur	26	30
Tibia	3	5
Humerus	7	5
Fibula	1	3
**METHOD OF SURGERY**		
Amputation	11	9
Limb salvage	26	34

*All factors were well balanced between two groups(p > 0.05)*.

In summary, these results demonstrated that OS patients with high DGKZ expression survived for shorter periods than those with low DGKZ expression and DGKZ might played an essential role in the development and progression of osteosarcoma.

### DGKZ Silencing Inhibited Growth and Promoted Apoptosis *in vitro*

**Figure 2:**
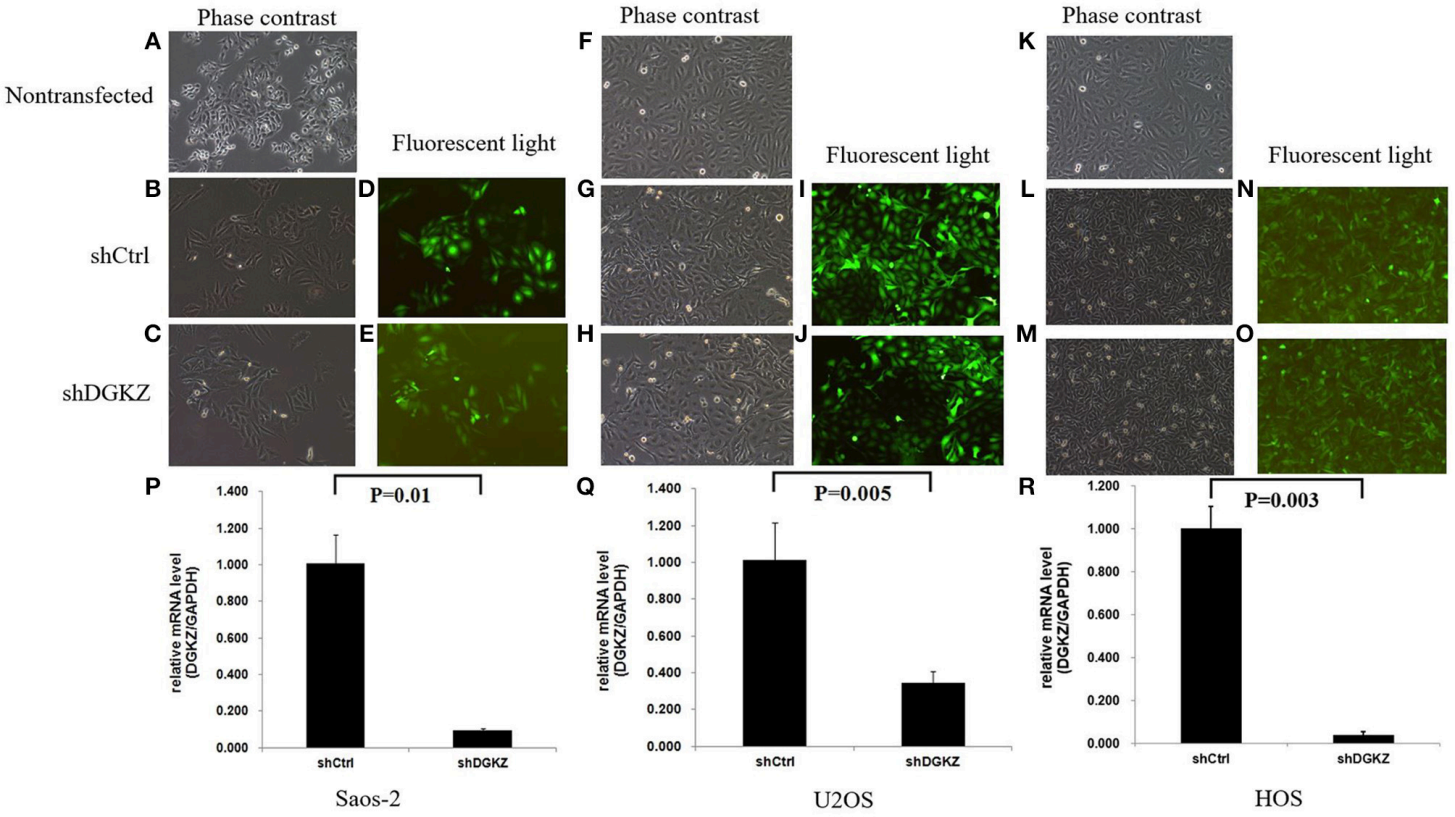
Lentivirus-mediated DGKZ knockdown specifically inhibited DGKZ expression in osteosarcoma cells: Saos-2, HOS, and U2OS infected with DGKZ -shRNA or shCtrl lentivirus and non-transfected cells examined by fluorescent microscopy and light microscopy at the 3rd day after infection. More than 80% of cells expressed GFP. There was no significant difference between the negative control group and the non-transfected group, indicating the transfection process has no effect on cells growth. **(A–C)** Saos-2, 100 × B; **(D,E)** Saos-2, 100 × G; **(F–H)** U2OS, 100 × B; **(I,J)** U2OS, 100 × G; **(K–M)** HOS, 100 × B; **(N,O)** U2OS, 100 × G; **(P–R)** Efficient knockdown of DGKZ in osteosarcoma cell lines. Saos-2 **(P)**,U2OS **(Q)**, and HOS **(R)** cells were transduced with lentivirus expressing control or DGKZ shRNA. The knockdown efficiencies were detected with qPCR.

According to the above result, we further explored DGKZ biological function through RNA interference. We did Lentivirus-mediated knockdown of DGKZ in Saos-2, U2OS and HOS cells. Fluorescent microscopy and light microscopy demonstrated that more than 80% of cells expressed GFP at the 3rd day after infection (Figures 2A–O). Compared with mock transfected cells, DGKZ's expression was significantly inhibited in cells with shRNA at mRNA level by qPCR analysis, which demonstrated that the lentiviral-based shRNA construct exerted a specific knockdown effect on endogenous DGKZ expression in Saos-2, U2OS, and HOS cells (Figures 2P–R).

**Figure 3:**
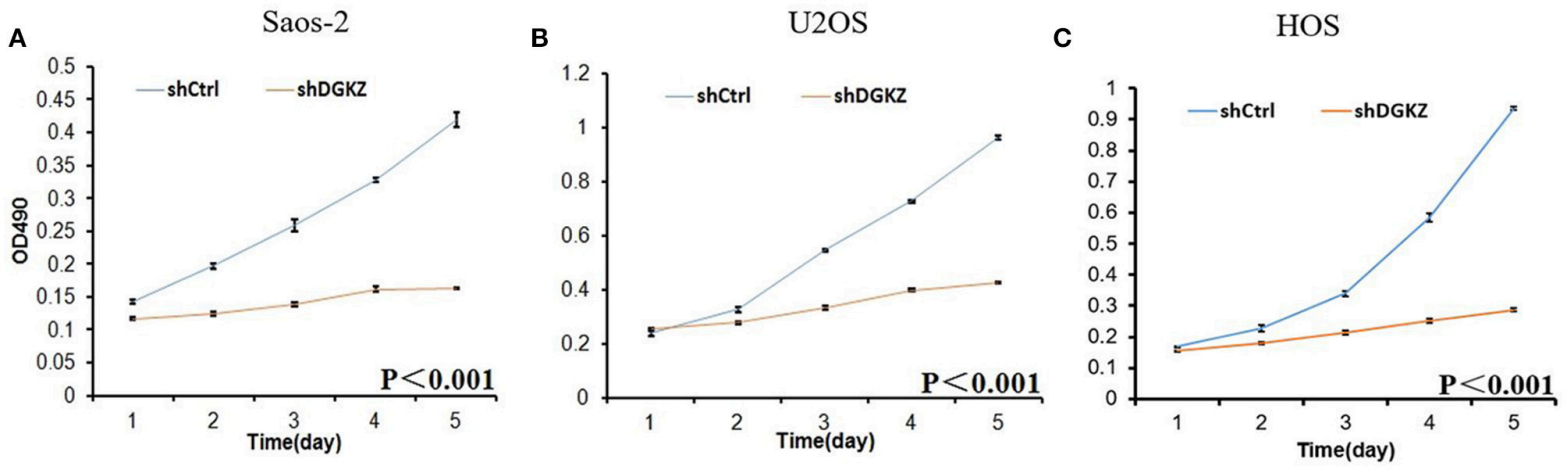
Knockdown of DGKZ inhibited cell proliferation ability in osteosarcoma cells by MTT assay: Saos-2 **(A)**, U2OS **(B)**, and HOS**(C)**.

**Figure 4:**
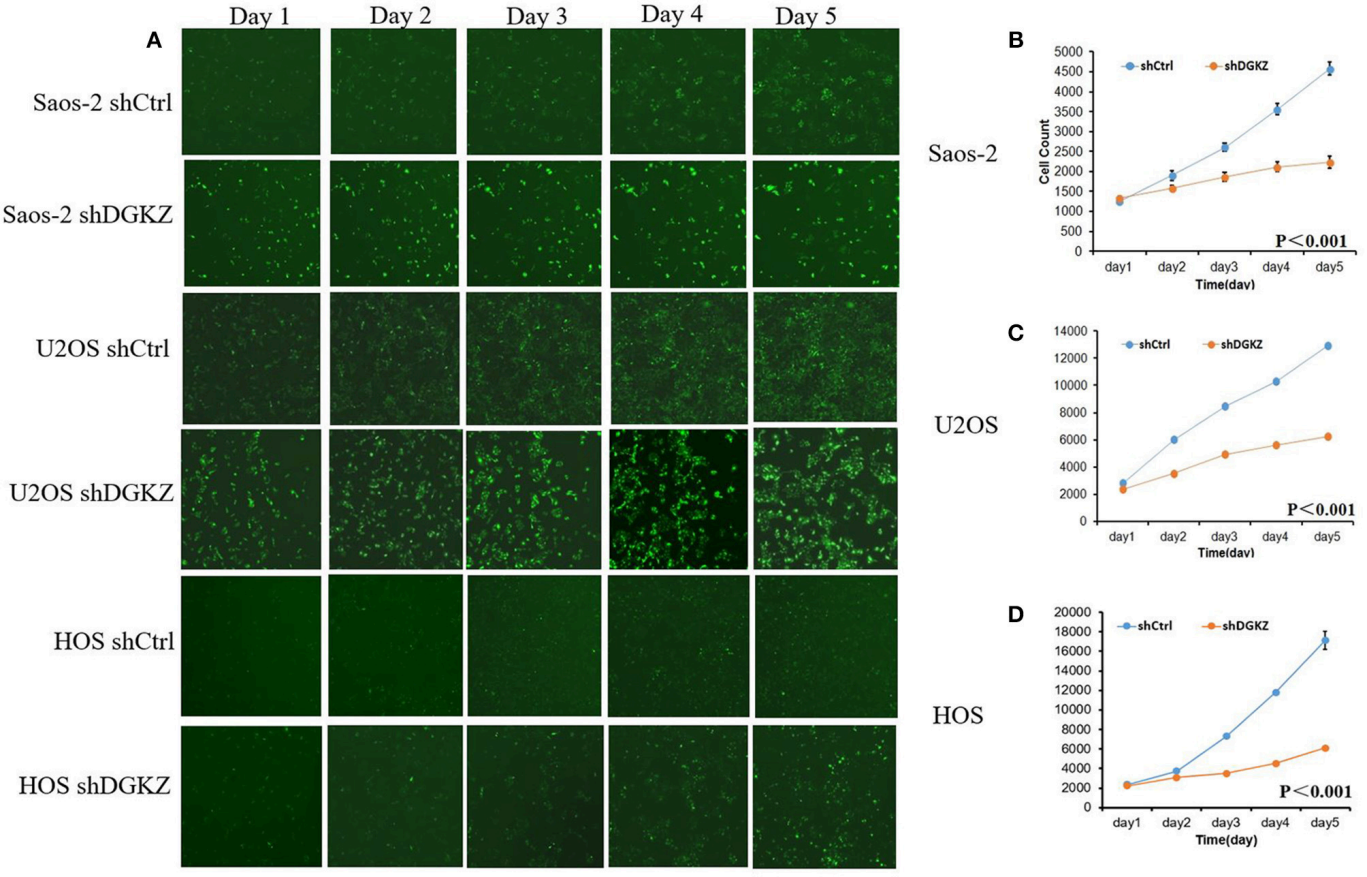
Knockdown of DGKZ inhibited cell proliferation ability in osteosarcoma cells by HCS assay: **(A)** Representative images of osteosarcoma cells with or without DGKZ-silencing continuously for 5 days; **(B–D)** HCS assay of cell proliferation of Saos-2 **(B)**, U2OS **(C)**, and HOS **(D)** cells.

Sustaining proliferation signaling is one of the core hallmarks of cancer (33). Thus, we investigated cell proliferation in Saos-2, U2OS, and HOS cells with or without shDGKZ by HCS and MTT assays. As shown in Figures 3, 4, significant decrease in proliferation was observed in shDGKZ group by MTT and HCS assay in all three cell lines.

**Figure 5:**
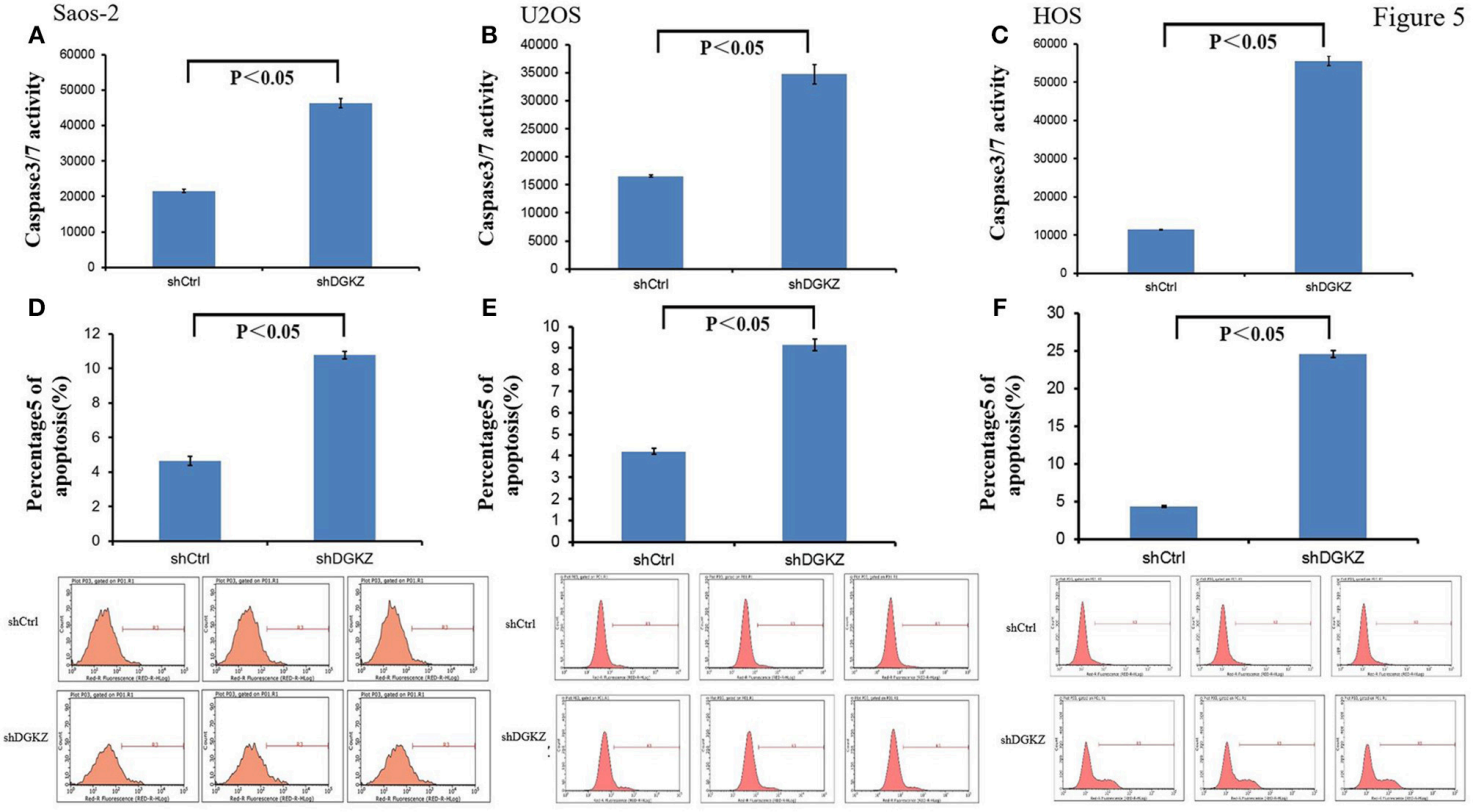
Knockdown of DGKZ promoted apoptosis in osteosarcoma cells: **(A–C)** Activation of caspase 3/7 was further increased in DGKZ-shRNA-treated osteosarcoma cells compared with control group in Saos-2 **(A)**, U2OS **(B)**, and HOS **(C)** cells. **(D–F)** Apoptosis was determined by flow cytometry assays in three osteosarcoma cell lines with DGKZ silence and control cells. The apoptotic rate was calculated as the percentage of Annexin FITC positive (**D**: Saos-2, **E**: U2OS, **F**: HOS).

Resisting cell death, another hallmark of cancers, is essential for tumors (34). Thus, we evaluated Caspase3/7 activity in Saos-2, U2OS, and HOS cells with shDGKZ. As demonstrated in Figures 5A–C, Caspase3/7 activity, measured by using a luminescent caspase activity assay kit, increased remarkably in DGKZ-silencing Saos-2/U2OS/HOS cells compared with control group. Annexin V-APC staining by FACS in Saos-2,U2OS and HOS cells following DGKZ -knockdown was also administrated to check impact of GINS1 on apoptosis. Figures 5D–F showed the apoptotic rate in DGKZ-silencing cells was much higher than that in control group in all three cell lines, suggesting that DGKZ knockdown facilitated apoptosis of osteosarcoma cells.

### Silencing of DGKZ Inhibits Tumorigenicity *in vivo*

**Figure 6:**
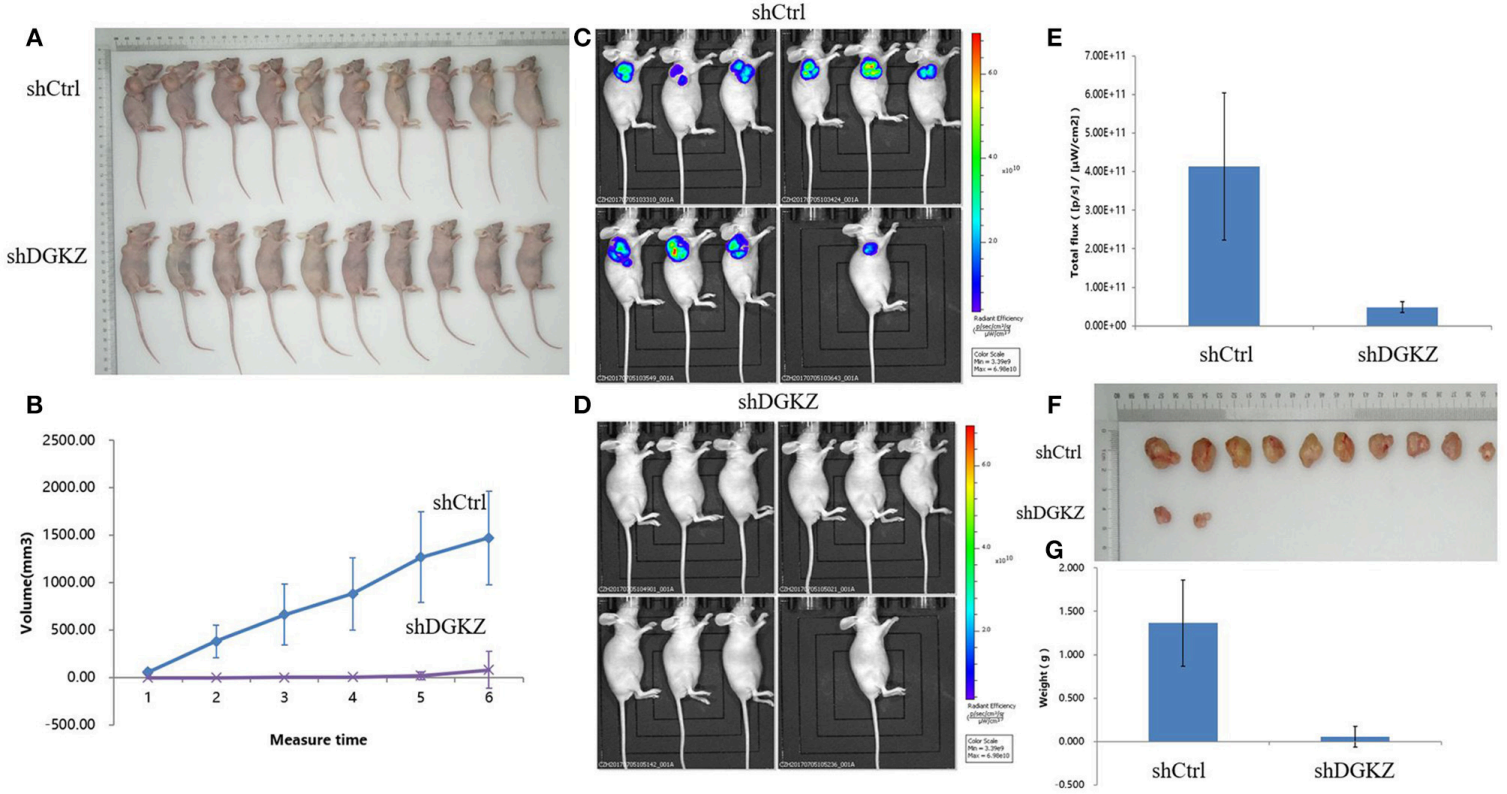
Knockdown of DGKZ suppresses tumourigenesis in an osteosarcoma xenograft nude mouse model: **(A)** Photograph shows the representative BALB/c nude mice bearing xenograft tumors formed by HOS osteosarcoma cells with or without DGKZ silencing in the same mice for comparison. **(B)** Growth curve of tumors in mice for duration of 4 weeks. DGKZ-knockdown formed significant smaller tumors than the control group (*n* = 10 for both). **(C)** Representative bioluminescent images of mice without DGKZ-silencing before sacrificed, as shown by intensive bioluminescent signals detected. **(D)** Representative bioluminescent images of mice with DGKZ-silencing before sacrificed, no bioluminescent signal was visualized. **(E)** Progression of BLI over time indicated significant difference between control group and DGKZ -knockout mice. **(F)** Photograph shows the dissected osteosarcoma tumors formed in host mice. **(G)** Significant smaller tumors were formed by DGKZ-silencing in xenograft.

We next examined whether DGKZ silencing in osteosarcoma affects OS cell growth *in vivo*. Right armpit region of BALB/c nude mice was chosen to perform subcutaneous injection with HOS cells with or without DGKZ-knockout. Volume of tumor was checked continuously after cell implantation under overall health status for all individuals. Tumors were removed and then subjected to measure weight. Consistent with our *in vitro* findings, DGKZ knockdown impaired proliferation (Figures 6A,B). Meanwhile, through bioluminescence measurement, as shown in the Figures 6C–E, the control group showed steeper progression compared with the DGKZ-silenced group. After scarification, the averaged tumor weight of the control mice was extremely heavier than that in DGKZ-silenced group which presented suppressed tumor growth (Figures 6F,G). These results indicated that knockout of DGKZ inhibited tumor growth of osteosarcoma *in vivo*.

### Gene Expression Profile Analysis Revealed MYC Pathway Was a Downstream Target Affected by DGKZ

**Figure 7:**
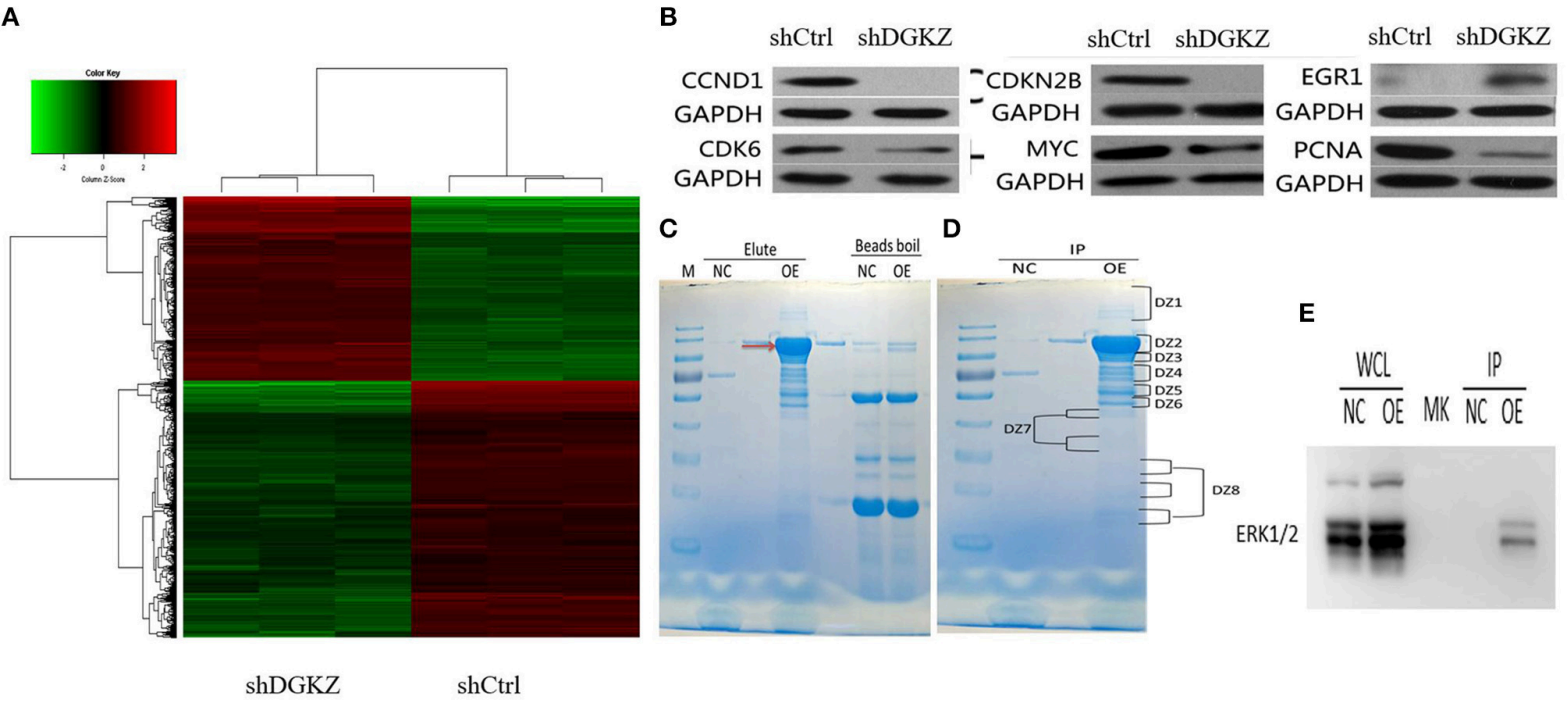
Effects of DGKZ knockdown on downstream gene expression according to microarray assay and identification of DGKZ's interaction protein through LC-MS/MS analysis: **(A)** Heatmap representation of genes significant differential expressions in HOS cells infected with lentivirus expressing shCtrl or shDGKZ under the criteria *p* < 0.05 and | fold change | >1.5. Genes and samples were listed in rows and columns, respectively. A color scale for the normalized expression data was shown at the bottom of the microarray heatmap (green represents downregulated genes while red represents upregulated genes). **(B)** Validation of downstream gene target expression in MYC pathway by Western blot. **(C)** The 3 × FLAG-DGKZ interacting complex was purified using the anti-FLAG magnetic beads (Red arrow: 3 × FLAG-DGKZ). In contrast to the control (sample pulled-down from control cells), the bands only in sample pulled-down from 3 × FLAG-DGKZ cells were excised for the following in-gel trypsin digestion(NC: negative control, OE: over expression). **(D)** The gel band was cut into pieces and washed with Milli-Q water. The tryptic peptides were extracted from the gel pieces and lyophilized by vacuum centrifugation. **(E)** Through LC-MS analysis and further co-immunoprecipitation/ immunoblotting, interaction between ERK1/2 and DGKZ was confirmed in HOS cells.

The previous data suggested that DGKZ played a key role in osteosarcoma growth. However, mechanisms underlying DGKZ-regulation are still unclear. Thus, Affymetrix GeneChip and IPA were administrated to display an overview of DGKZ possible biological interaction through DGKZ knockdown in HOS cells. Differentially expressed genes with at least a 1.5-fold change were identified. As shown in Figure 7A, 807 genes were up-regulated and 1,110 genes were down-regulated in DGKZ knockdown HOS cells compared with negative control cells. Ingenuity Pathway Analysis (IPA) revealed that several functional classifications were significantly enriched, including cell assembly/organization, cell death/survival, organismal survival, cell cycle, cellular function/maintenance, cellular development, cellular growth/proliferation, cellular movement, tumor morphology, and cell signaling (Supplemental Figure 1). Further, IPA in “canonical pathway” module revealed that several critical pathways involved in cancer development and apoptosis such as “ILK signaling,” “signaling by Rho Family GTPases,” and “BMP signaling pathway” were activated while pathways such as “RhoGDI signaling” was inhibited by DGKZ knockdown(Supplemental Figure 2). These data demonstrated that the effects of DGKZ in osteosarcoma progression were likely dependent on regulation of several key cellular functions.

Additionally, selected gene lists obtained from the microarray analyses were uploaded to IPA system and a core biologic pathway analysis was performed to identify molecular networks. Ingenuity Pathway Analysis (IPA) revealed that after DGKZ knockdown, target genes expression in MYC pathway were altered(Supplemental Figure 3), including CCND1, CDKN2B, CDK6, MYC, PCNA, and EGR1, which were further confirmed by Western blot (Figure 7B). Taken together, these data imply that role of DGKZ in osteosarcoma pathogenesis may function via regulation of MYC signal pathway.

### Identification of the ERK1/2 as DGKZ Interacting Proteins in MNNG/HOS Cells

Co-immunoprecipitated proteins were separated using SDS-PAGE and stained (Figure 7C). Coomassie staining of the gels loaded with DGKZ-IP identified several bands that were not present in vector or IgG control. Immunoprecipitated proteins were gel extracted, trypsin digested, and identified by LC-MS/MS analysis (Figure 7D). We identified 100 unique proteins with a protein False Discovery Rate equal or lower than 1%. We then applied a manual thresholding approach and a probabilistic PPI prediction algorithm to compute the most likely associations between each of these 100 proteins and DGKZ. Followed by co-immunoprecipitation and immunoblotting, we identified ERK1/2, a key component in Ras/Raf/MAPK pathway and a key MYC-interactor, as a candidate protein interacted with DGKZ in HOS cells (Figure 7E).

## Discussion

Osteosarcoma is the most common type of primary malignant bone tumor affecting both children and adults (35). Even the outcome for patients with osteosarcoma has improved dramatically by therapeutic strategies, those who suffered from distant metastasis or local recurrence continue to pose a particularly tough challenge (3). Thus, a better understanding of the molecular biology of OS is needed and may improve therapeutic efficiency. Diacylglycerol kinase zeta (DGKZ) has proven to be involved in several physiological or pathophysiological signaling pathways, including certain human carcinogenesis. In our study, we speculated that DGKZ acts as an oncogene in proliferation of osteosarcoma. Expression of DGKZ was significantly up-regulated in OS tumor samples compared with normal bone tissue, and was associated with poor prognosis. Moreover, *in vitro* studies, through a constructed lentivirus expressing DGKZ-specific shRNA, we further demonstrated the key role of DGKZ in cell growth and apoptosis in Saos-2/U2OS/HOS cells. Additionally, considering that a suitable animal model was needed to investigate the effect of DGKZ *in vivo*, we chose BALB/c nude mice, one of the most widely used models, to establish xenograft model induced by subcutaneous injection of HOS cells. Consistent with findings *in vitro*, our data also strongly supported the positive correlation between DGKZ expression and osteosarcoma growth. Taken together, these data indicated substantial evidence that DGKZ is a prognostic marker and potential new target for cancer therapy in osteosarcoma.

Previous data demonstrated that DGKZ was involved in tumor initiation and progression (26–28). Cai et al. (27) found that high DGKZ expression could facilitate Rho GTPase activation and promote motility of metastatic function in colon cancer. Diao et al. (28) demonstrated positive correlation between DGKZ expression and gliomagrade in glioma tumor samples. Furthermore, DGKZ knockdown in human glioma suppressed cell proliferation, hampered colony formation ability, and promoted cell cycle arrest and apoptosis. Torres-Ayuso et al. (26) explored the potential molecular mechanism of DGKZ's role in rapamycin-resistant colon cancer cell line, and found that DGKZ manipulated mTORC1 and lipogenic metabolism in colon cancer cells through SREBP-1. In line with these data, we also proved that role of DGKZ acts an oncogene in proliferation of osteosarcoma.

The regulation of DGKZ on downstream signal pathway in cancer cells is still not well-elucidated. To investigate the potential mechanism about DGKZ in regulating tumorigenesis of osteosarcoma, we conducted microarray analysis to explore alteration of cancer-related genes between normal osteosarcoma cells and DGKZ knockdown cells. Microarray data demonstrated that DGKZ's oncogene role is the result of regulating a number of different signaling pathways, which is consistent with a biofunctional role for DGKZ reported in previous study (16, 18–25). IPA analysis with confirmed western blot data demonstrated that DGKZ siRNA-expressing cells posed a downregulated activity of MYC signal pathway and an altered expression of several target genes in MYC pathway. MYC proto-oncogene promotes oncogenic transcriptional amplification program in cancers and represents as an important therapeutic target for cancer therapy. In addition, misregulated expressions of MYC are often associated with osteosarcoma oncogenesis and progression (36). Taken together, it could be concluded that DGKZ might exert an enhancing effect on activity of MYC signal pathway to impact on osteosarcoma proliferation.

The MYC signaling pathway consists of a family of upstream and downstream targets that are associated with cell proliferation and growth. Whether regulation of DGKZ on downstream MYC pathway in osteosarcoma is through a direct or indirect route is still not clear. In our study, through IP-MS analysis, we provided potential evidence that the interaction of ERK1/2 and DGKZ was closely linked to MYC-dependent osteosarcoma development. ERK1/2 are widely expressed in various cells including cardiomyocytes, neurons, and hepatocytes. They are directly activated by phosphorylation of mitogen-activated protein kinase (MAPK) to promote cellular differentiation, proliferation, and survival (37). In pathological situation, such enhanced/constitutive activation of ERK1/2 existed in various human malignancies, including osteosarcoma (38–40). Various articles have showed activation of the ERK-pathway prolonged the half-life of the MYC protein and thus enhances the accumulation of MYC activity, both in normal cell growth control and oncogenesis (41, 42). This finding enriched our knowledge on how DGKZ exerted an impact in osteosarcoma MYC-dependent proliferation. Thus, we speculated that DGKZ might regulate MYC signal pathway via possible interaction with ERK1/2 in carcinogenesis of osteosarcoma and utilization of DGKZ-ERK1/2-MYC interacting axis inhibitor might be an effective approach in treatment of osteosarcoma. However, the detailed mechanism of MYC pathway regulation induced by DGKZ-ERK1/2 needs further investigation.

In conclusion, our study demonstrated that DGKZ, a potential prognostic and predictive indicator in osteosarcoma, play a key role in proliferation of osteosarcoma, via its possible interaction with ERK1/2 and regulation of MYC pathway. These data provide an important starting point for future studies investigating the potential of DGKZ-ERK1/2-MYC axis as a potential new target for cancer therapy in osteosarcoma.

## Author Contributions

WY drafted the manuscript, participated in the design of the study, data collection. and analysis. LT and FL participated in data collection and analysis. YY was involved in drafting the manuscript. ZS designed the study and helped to draft the manuscript. All authors read and approved the final manuscript.

### Conflict of Interest Statement

The authors declare that the research was conducted in the absence of any commercial or financial relationships that could be construed as a potential conflict of interest.

## Supplementary Material

Supplemental Figure 1 DGKZ related disease and function enrichment was analyzed based on IPA databases.

Supplemental Figure 2 DGKZ related functional pathway enrichment was analyzed based on IPA databases.

Supplemental Figure 3 Up-stream and down-stream network of MYC analyzed by IPA.
