# Supplementary figures and images for: DGKZ Acts as a Potential Oncogene in Osteosarcoma Proliferation Through Its Possible Interaction With ERK1/2 and MYC Pathway
Source: Front Oncol. 2019 Jan 4;8:655. doi: 10.3389/fonc.2018.00655 (PMC6328465; doi:10.3389/fonc.2018.00655)

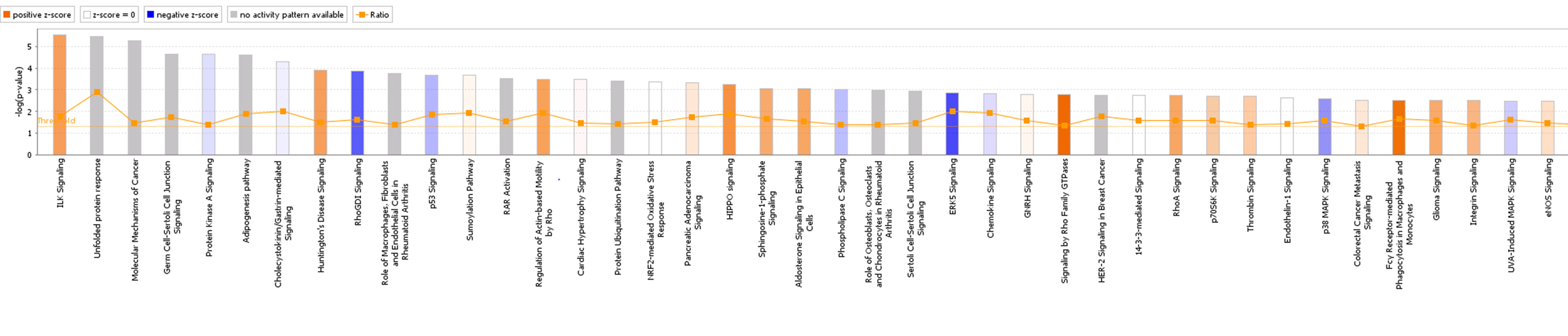

Supplement: Supplemental Figure 1 — DGKZ related disease and function enrichment was analyzed based on IPA databases. [file Image_1.TIF]

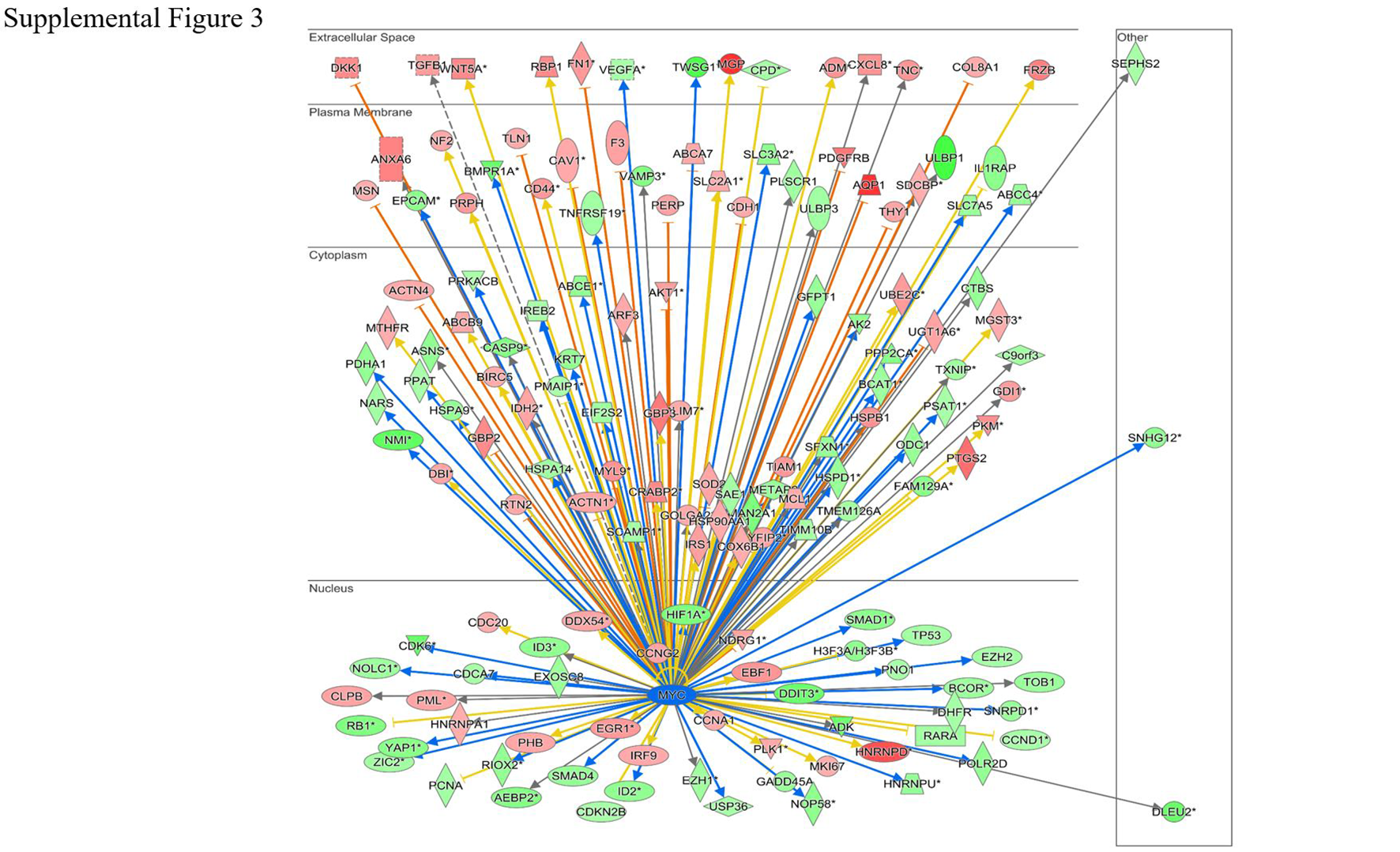

Supplement: Supplemental Figure 3 — Up-stream and down-stream network of MYC analyzed by IPA. [file Image_3.TIF]
